# Effects of pandemics uncertainty on fertility

**DOI:** 10.3389/fpubh.2022.854771

**Published:** 2022-08-30

**Authors:** Yonglong Wang, Giray Gozgor, Chi Keung Marco Lau

**Affiliations:** ^1^Research Centre of Modern Economic and Management, Zhejiang Yuexiu Univerisity of Foreign Languages, Shaoxing, China; ^2^School of Management, University of Bradford, Bradford, United Kingdom; ^3^Faculty of Political Sciences, Istanbul Medeniyet University, Istanbul, Turkey; ^4^Adnan Kassar School of Business, Lebanese American University, Beirut, Lebanon; ^5^Department of Economics and Finance, The Hang Seng University of Hong Kong, Shatin, Hong Kong SAR, China

**Keywords:** the COVID-19 pandemic, fertility behavior, pandemics uncertainty, uncertainty shocks, World Pandemics Uncertainty Index (WPUI)

## Abstract

The COVID-19 pandemic has affected various dimensions of the economies and societies. At this juncture, this paper examines the effects of pandemics-related uncertainty on fertility in the panel dataset of 126 countries from 1996 to 2019. For this purpose, the World Pandemics Uncertainty Indices are used to measure the pandemics-related uncertainty. The novel empirical evidence is that pandemics-related uncertainty decreases fertility rates. These results are robust to estimate different models and include various controls. We also try to explain why the rise in uncertainty during the COVID-19 pandemic has resulted in the fertility decline.

## Introduction

The COVID-19 pandemic has affected various aspects of the economy and society. It has led to different fiscal and monetary policy implications (1, 2), which also caused higher price volatility in assets, commodities and financial markets [see, e.g., (3, 4)]. It is shown that the uncertainty shocks related to the pandemic can distort the current capitalist economic system. For instance, pandemics and household consumption have decreased global economic activity (5–8). The pandemics have also increased income inequality (9) and conflicts (10).

Fertility is also affected by cultural, economic, political, and social factors (11). Previous papers have observed that fertility negatively correlates with economic development in the long run [see, e.g., (12–14)]. The demographic change can also affect the demand side of the economy and thus the economic performance. It can also provide implications for population aging, retirement, and social welfare. In this paper, we aim to examine the effects of pandemic-related uncertainty on the fertility rate.

It is also important to note that uncertainty shocks can also be essential in fertility behavior. Expectations related to business cycles can affect the change in fertility rates. For instance, in a recent empirical study on more than 100 million births in the United States from 1988 to 2014, Buckles et al. (15) observed that fertility decision is forward-looking. It is related to the short-run expectations about employment and income changes. Therefore, individuals can react to uncertainty shocks and change short-run expectations. These uncertainty shocks can come from the news, stock markets and other macroeconomic indicators. In this paper, we also suggest that pandemics-related information can also change the short-run expectations of households, and this issue can affect their fertility decisions. A higher level of “bad news” related to the pandemics can create pessimistic expectations of future incomes (wages) and the stability of the current jobs or finding new jobs. The expectations during pandemics can lead to declining consumer confidence and reduced household consumption. This effect is known as the precautionary savings motivation in the literature (16). There are other concepts of uncertainty in social sciences [see, e.g., (17)]. For instance, social distancing and the fear of contagion also changed people's behavior during the pandemic (18–20). Similarly, staying indoors during the pandemic can also affect pregnancy plans.

All in all, families can postpone having a child during periods of higher uncertainty (21). We suggest that precautionary savings explain countries' cross-country fertility levels and changes. Several theoretical models link precautionary savings to fertility decisions [e.g., (22)]. However, it is essential to note that empirical studies on testing the validity of precautionary savings motivation have provided mixed findings.

In this paper, we assume that pandemic-related uncertainty by a decline in consumer confidence and reduced or delayed household consumption–suggesting that “precautionary saving motivation” is the primary mechanism affecting fertility plans during the pandemic. However, we can also indicate that becoming pregnant does not have to immediately increase or affect consumption; instead, having a child influences family consumption and spending over a very long period when the child is growing up and in education. In addition, our precautionary saving perspective is reducing the potentially wide-ranging impact of uncertainty just to one specific factor, ignoring many other relevant motivations and potential drivers of fertility decisions, including the actual experiences of economic hardship (unemployment, loss of income, unstable employment), health consequences of infection or limitations due to lockdowns and government measures intended to limit the spread of the COVID-19. For instance, women in some Latin American countries were advised not to get pregnant during the Zika Epidemic as the disease could damage the fetus's brain during pregnancy. Thus, precautionary saving should be seen as one of many possible factors affecting fertility due to pandemic-related uncertainty.

This paper aims to contribute to the empirical literature by examining the effects of pandemics-related uncertainty on fertility behavior. We focus on precautionary savings motivation to explain the level of fertility rate and changes. At this point, we use a novel measure of pandemics-related uncertainty, so-called the World Pandemics Uncertainty Index (WPUI), proposed by Ahir et al. (23). The WPUI was created by focusing on country reports of the Economist Intelligence Unit. The country reports trackback to economic agenda, policy implications, and political aspects to model uncertainty shock about the pandemics conditions in a given country. Indeed, events and policies related to pandemics can be defined as exogenous shocks. In other words, pandemic-related uncertainty should affect fertility behavior (24). The empirical results from the panel dataset of 126 countries from 1996 to 2019 show that pandemics-related uncertainty decreases the fertility rate. These results are robust to estimating different models. We suggest that this evidence may also explain why the rise in anticipation during COVID-19 has resulted in fertility decline in various countries.

The rest of the paper is organized as follows. Section Literature review on fertility decisions reviews the previous articles in the literature. Section Empirical model and data sets the empirical model and provides the features of the data. Section Empirical results discusses the empirical findings for the countries at different stages of economic development. Section Robustness checks provides the results of the robustness analyses based on other empirical models. Section Conclusion and recommendations concludes.

## Literature review on fertility decisions

The fertility theory documents a negative association between fertility and economic development in the long run. Specifically, fertility rates decline as a country develops (11, 13, 25). However, the direction of the relationship between fertility and economic growth is positive in the short run (26). Therefore, fertility decisions are sensitive to business cycles, making fertility a pro-cyclical indicator (15). Therefore, we need to separate fertility rates' long-run and short-run drivers. For this purpose, we use the level of fertility rates and the differences in the fertility rates for modeling long-run and short-run effects, respectively.

In microeconomics, the marginal utility of consumption is convex (27, 28). This issue explains the validity of the “precautionary” savings. According to this view, uncertainty increases precautionary savings by reducing current consumption and lowering fertility (16, 21). Therefore, a higher uncertainty (such as pandemics) will increase the motivation for precautionary savings and cause lower fertility in a country. Pandemics may not fully explain the cross-country differences between fertility rates. Still, this issue should be valid in the short run as the pandemics should affect business cycles.

### Determinants of fertility rates

Many studies have provided evidence of the negative impact of human capital on fertility rates in the long run (29–32). Early studies, such as Becker (33, 34) and Becker and Lewis (35), show the significant impact of human capital on fertility behavior. According to these models, the increasing education level (human capital) leads to higher costs. The rationale behind the effect is that as education increases, children will elderly join the labor market, decreasing the income of their parents' families due to their higher expenditures for their children's education. Another reason is that parents with higher education will spend their time on full-time work. Therefore, they will decide to have fewer children. Thus, in a higher level of human capital, the quality of the child, rather than quantity, will be prefered (14). Women's employment and roles, changing values and aspirations also have crucial impacts on fertility postponement due to the incompatibility of education enrolment and parenthood (13).

There is also a negative theoretical relationship between the fertility rate and per capita income. According to Greenwood and Seshadri (36), the negative relationship between fertility and per capita income comes from the structural transformation from agriculture to manufacturing and services. Other theoretical approaches, provided by Barro and Becker (37), Boldrin and Jones (38), Ehrlich and Kim (39), Kalemli-Ozcan (40), and Sah (41), indicate that as the country develops then, infant and child mortality reduces. Therefore, fertility will decline as the country reaches a higher per capita income [e.g., (42–44)].

In addition, Ehrlich and Kim (39), Alhassan et al. (45), Evans et al. (46), Finlay (47), Nandi et al. (48), and Nobles et al. (49) find that life expectancy affects fertility rates. According to most of these studies, health developments can increase life expectancy and boost economic growth and fertility rates. However, improved life expectancy can also suppress fertility rather than improve it. Women need to give birth to fewer children. It is important to note that Hoem (50) criticized a strong mechanistic focus on reporting and discussing significance in fertility modeling.

Following these discussed papers, we should include economic development, human capital, and life expectancy as the main controls in the empirical models for the long run.

### Effects of uncertainty shocks on fertility rates

There are also several determinants of the changes in the fertility rates, which are mainly related to short-run business fluctuations. Uncertainty is one of the leading determinants of fertility changes, given that consumption and income uncertainty decrease fertility rates. According to the precautionary motivation approach, uncertainty in income leads to pessimist expectations. It decreases “consumer confidence” during recessions (15). Consumer confidence is negatively associated with fertility, given a higher level of economic uncertainty (51). Shocks related to natural disasters or pandemics can increase health concerns over having children. For instance, Gozgor et al. (51), Alderotti et al. (52), Chabe-Ferret and Gobbi (53), Comolli and Vignoli (54), Hanappi et al. (55), Hondroyiannis (56), Matysiak et al. (57), and Sobotka et al. (58) find that a higher uncertainty decreases fertility rates. However, Kohler and Kohler (59) and Kreyenfeld (60) indicate no significant relationship between uncertainty and fertility rates during the period following the breakdown of the Soviet Union and the state-socialist political system in Central and Eastern Europe. De la Croix and Pommeret (22) theoretically explain the clashing evidence in the empirical literature. The authors indicate that there could be a reverse causality: fertility decisions affect uncertainty, i.e., employed women (or parents) face more substantial labor market uncertainty (61, 62).

Previous empirical papers suggest finding a “purely exogenous” measure of uncertainty to investigate the relationship between uncertainty and fertility. We offer that the WPUI is a purely exogenous indicator to measure uncertainty.

### Epidemics, pandemics, and fertility rates

Several papers have focused on the effects of the COVID-19 pandemic on fertility rates. However, previous articles focus on the impact of the earlier pandemics before COVID-19. For instance, Boberg-Fazlic et al. (63) document that the 1918–9 Spanish Flu pandemic decreases the fertility rate in Sweden in the long run. Similar evidence is obtained by Chandra et al. (64) for the states level data in the United States. Marteleto et al. (65) observed that the Zika Epidemic significantly reduced Brazil's fertility rate in 2016.

Regarding the COVID-19 studies, Aassve et al. (66) discuss that the impact of the COVID-19 pandemic on fertility depends on the countries' income levels. Economic uncertainty related to COVID-19 should decrease the fertility rate in high-income economies. Still, the relationship can be mixed in the low-income and middle-income economies due to the role of informal economies. In a further study, Aassve et al. (67) found that the COVID-19 pandemics decrease fertility rates. The most significant declines are observed in Italy, Spain, and Portugal.

On the other hand, Ullah et al. (68) discuss how COVID-19 affects future birth rates, which are expected to be negative. Voicu and Bădoi (69) theoretically show that the COVID-19 pandemic significantly affects fertility decisions due to economic uncertainty, health emergency, and social distancing measures. Fostik (70) also reviews the previous papers and indicates that COVID-19 is expected to decrease the fertility rate in Canada. Berrington et al. (71) document that the COVID-19 pandemic is negatively associated with the fertility rate in the United Kingdom. Luppi et al. (72) observe that COVID-19 reduces fertility plans in young people (18-34 years old) in Germany, France, Spain, and the United Kingdom. Ghosh (73) also finds that the COVID-19 pandemic decreases the fertility rate in Hong Kong and South Korea.

Furthermore, Rovetta (74) demonstrates that the Google Trends data accurately captures the anomalies related to the COVID-19 pandemic in Italy's regions. Similarly, Wilde et al. (24) consider the Google Trends data and predict that the fertility decline due to the COVID-19-related uncertainty in the United States would be 50% higher than the fertility decline due to the Global Financial Crisis 2008-9. However, Berger et al. (75) also use the Google Trends data and find that the COVID-19-related economic uncertainty has little impact on fertility changes in the United States.

Following the precautionary motivation hypothesis, we focus on the uncertainty related to pandemics, which can affect fiscal, monetary, and other policy changes. Indeed, pandemics have significantly affected the world economy *via* declining trade volumes and portfolio flows. Pandemics also have created uncertainty over future income, affecting the expectations similar to business cycles. We aim to contribute to the empirical literature by connecting pandemics-related uncertainty and the fertility rate. Following De la Croix and Pommeret (22), we define the pandemics-related uncertainty as exogenous to fertility rates.

## Empirical model and data

### Empirical models

We focus on the panel dataset of 126 countries from 1996 to 2019. The list of countries is provided in Appendix Table A1. The data capture the countries at the different stages of economic development. Therefore, we also split the countries into the Organization for Economic Co-operation and Development (OECD) and non-OECD countries. Following previous papers, we aim to explain cross-country differences in the level and the change in fertility rate over the period under concern. We, therefore, use the level and the changes (first differences) in the WPUI across 126 countries. We also control for the lagged fertility, the lagged per capita GDP, the lagged human capital, the lagged life expectancy, and the measures of the WPUI. At this stage, we estimate the following equations:


(1) $$\begin{array}{l} \Delta Fertility_{i,t}=\alpha_{0}+\alpha_{1}\;WPUI_{i,t-k}+{\alpha_{2}\;X}_{i,t-1}+\;\vartheta_{i,t}\;{+\;\varepsilon }_{i,t} \end{array}$$

(2) $$\begin{array}{l} \Delta {Fertility}_{i,t}=\beta_{0}+\beta_{1}\;\Delta {WPUI}_{i,t-k}+{\beta_{2}\;X}_{i,t-1}+\;\vartheta_{i,t}\;{+\;\varepsilon }_{i,t} \end{array}$$

(3) $$\begin{array}{r} \Delta Fertility_{i,t}=\gamma_{0}+\gamma_{1}\;Fertility_{i,t-1}+\gamma_{2}\;WPUI_{i,t-k}\\ +{\gamma_{3}\;X}_{i,t-1}+\;\vartheta_{i,t}\;{+\;\varepsilon }_{i,t} \end{array}$$

(4) $$\begin{array}{r} \Delta Fertility_{i,t}=\delta_{0}+\delta_{1}\;Fertility_{i,t-1}+\delta_{2}\;\Delta {WPUI}_{i,t-k}\\ \;+{\delta_{3}\;X}_{i,t-1}+\;\vartheta_{i,t}\;{+\;\varepsilon }_{i,t} \end{array}$$

In Equations from (1) to (4), Δ*Fertility*_*i, t*_, *Fertility*_*i, t*_, and *Fertility*_*i, t*−1_ are the first difference, the level of current and the level of lagged fertility rate in country *i* and time *t*. *WPUI*_*i, t*−*k*_ and Δ*WPUI*_*i, t*−*k*_ are the World Pandemics Uncertainty Index in country *i* at time *t-k*. *X*_*i, t*−1_ represents the vector for control variables. Finally, ϑ_*i, t*_, and ε_*i, t*_ represent the “time and cross-section fixed-effects” and “error terms”. We estimate these models using the fixed-effects estimations, the standard estimator in the empirical papers.

The dependent variables are the level and the change in total fertility rate (births per woman), downloaded from World Bank (76). Some models have also included lagged fertility, which can model unobservable determinants, such as culture and religion, to affect fertility behavior. The income effect and economic development level are captured by the lagged log of per capita GDP (constant 2010$ prices) in terms of control variables. The data are obtained from World Bank (76). Total life expectancy at birth (years) is also included in the models. Life expectancy captures the public health conditions, which are also correlated with child mortality (77) and parents' health conditions (78). The data are also downloaded from World Bank (76). Finally, the index of human capital, based on the stock measure of years of schooling, is also used. The human capital index is based on the educational attainment data of Barro and Lee (79) and the quality of education. Human capital has captured the knowledge on the cost of children, and the related data are accessed from the Penn World Table (PWT) (version 10) in Feenstra et al. (80).

Table 1 provides a summary of the descriptive statistics of all these variables.

**Table 1:** Descriptive statistics.

**Indicator**	**Definition**	**Data source**	**Mean**	**Std. Dev**.	**Min**.	**Max**.	**Obs**.
Total fertility rate	Births per woman	World Bank (76 )	3.107	1.655	0.901	7.715	3,266
Per capita GDP (constant 2010 US$)	Logarithmic form	World Bank (76)	8.341	1.545	5.233	11.43	3,377
Life expectancy at birth	Total (years)	World Bank (76)	68.45	9.984	35.38	84.93	3,266
Human capital	Index	PWT 10.0, Feenstra et al. (80)	2.437	0.718	1.053	4.351	3,024
World pandemics uncertainty index (WPUI)	Index	International Monetary Fund, Ahir et al. (23)	0.106	1.417	0.000	56.47	3,408

**Figure 1:**
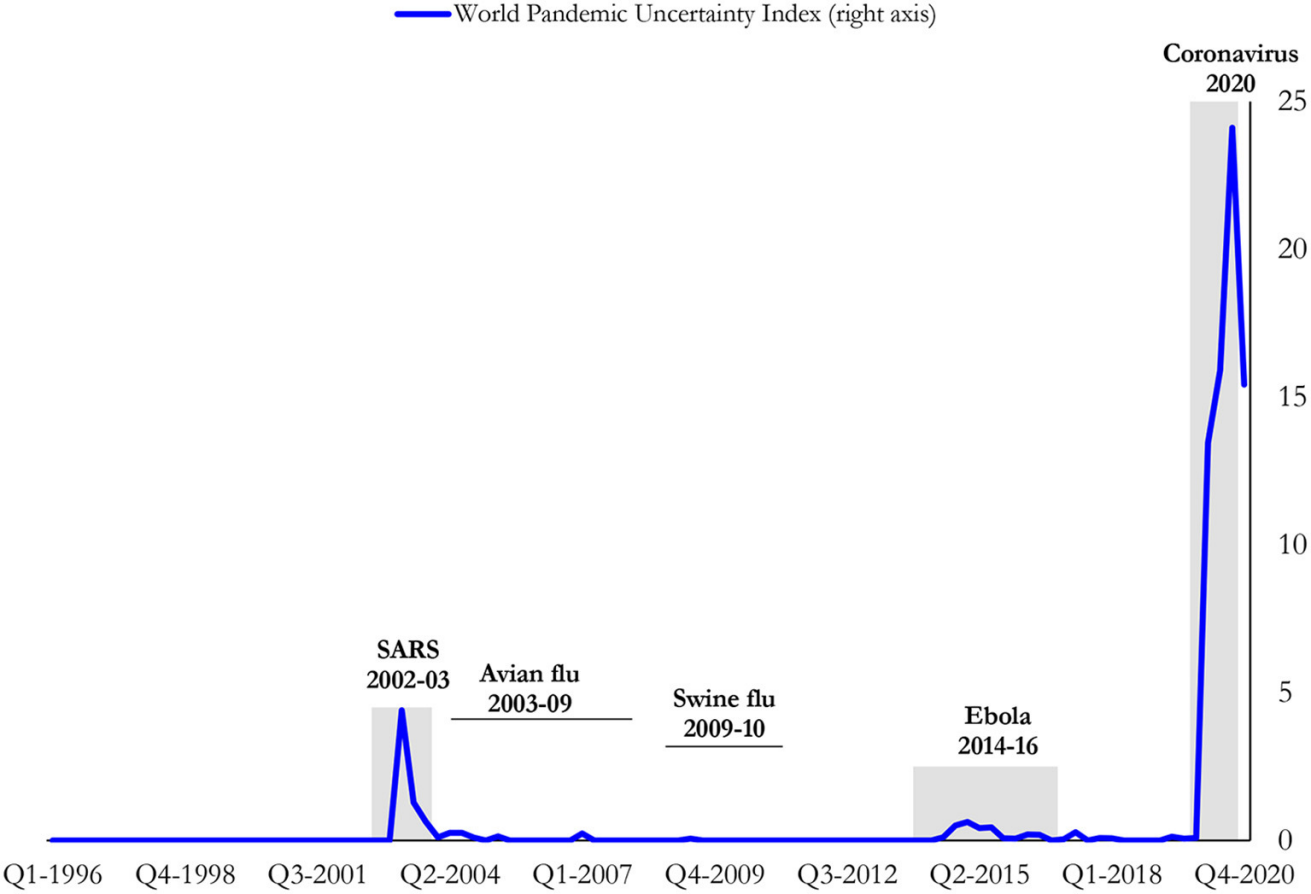
World pandemics uncertainty index (WPUI) (1996–2021). Data Source: https://worlduncertaintyindex.com/data/, provided by Ahir et al. (23).

The primary variable of interest is the level and the change of the WPUI in the lagged and the current forms. The related data have been accessed on the Ahir et al. (23) introduced by their website. The index is constructed by text mining from the country reports of the Economist Intelligence Unit. The index is based on counting words related to “pandemics”, the total words in articles and country reports. The WPUI significantly changes across countries, and the variations are determined by unpredictable pandemics-related shocks (23). The WPUI properly reflects the threat of pandemics since it aims to systematically assess the local pandemic conditions. The main advantage of the WPUI is that it is a comparable measure across countries, but it is limited to 142 countries. Several studies also use this indicator [e.g., (8)]. During the period between 1996 and 2019, most of the world regions had been free from pandemics; however, the WPUI has increased during the periods of several pandemics, such as the Avian Flu, Bird Flu, Ebola, Influenza, the Middle East Respiratory Syndrome (MERS), the Severe Acute Respiratory Syndrome (SARS), and Swine Flu. The WPUI is used for 126 countries in the empirical analyses. The frequency of the data is annual, which is the average of the quarterly data. The starting date is 1996, which is related to the data availability. Furthermore, Figure 1 provides a graph of the WPUI, the average values of countries, according to their GDPs from 1996 to 2021.

## Empirical results

### Level of pandemics-related uncertainty shocks

Table 2 provides the results of the fixed-effects estimations for Model (1), which focus on the difference between the fertility rate and the level of the WPUI.

**Table 2:** Fixed-effects estimations for Model I (1996–2019).

**Regressors**	**All countries**	**All countries**	**Non-OECD**	**Non-OECD**	**OECD**	**OECD**
Log per capita GDP_t−1_	0.022*** (0.007)	0.023*** (0.007)	0.021** (0.008)	0.021** (0.008)	0.054*** (0.017)	0.054*** (0.017)
Life expectancy_t−1_	−0.003*** (0.001)	−0.003*** (0.001)	−0.003*** (0.001)	−0.003*** (0.001)	−0.008*** (0.002)	−0.008*** (0.002)
Index of human capital_t−1_	0.091*** (0.017)	0.091*** (0.017)	0.104*** (0.020)	0.105*** (0.020)	0.054 (0.039)	0.053 (0.038)
WPUI_t_	−0.195*** (0.048)	–	−0.166*** (0.057)	–	−0.566 (0.523)	–
WPUI_t−1_	–	−0.296 (0.181)	–	−0.248 (0.165)	–	−1.555 (1.173)
Intercept	−0.204*** (0.064)	−0.204*** (0.064)	−0.216*** (0.065)	−0.217*** (0.066)	0.042 (0.155)	−0.045 (0.155)
Observation	2,722	2,722	2,024	2,024	748	748
Countries	126	126	92	92	34	34
*R*^2^ (Within)	0.109	0.109	0.156	0.157	0.031	0.031

The dependent variable is ΔFertility Rate_t_. The standard errors are in ( ).

^***^p < 0.01.

^**^p < 0.05.

Columns 1 and 2 report the results for all (126) countries from 1996 to 2019. The first column provides the findings of the current WPUI, and the second column provides the results of the lagged WPUI. In addition, the findings of 92 non-OECD economies are provided in Columns 3 and 4. The results of 34 OECD economies are reported in Columns 5 and 6.

In all countries, the coefficient of the current WPUI is −0.195, and it is significant at the 1% level. Similarly, the coefficient of the current WPUI is −0.166 for the non-OECD economies, and it is significant at the 1% level. However, the current WPUI is −0.566 for the OECD economies, statistically insignificant. Note that the coefficients of the lagged WPUI are positive, but they are statistically insignificant. These results indicate that the pandemics-related uncertainty has an adverse and temporary effect on fertility behavior in developing countries. This evidence may be explained that the non-OECD economies have less human capital than the OECD economies. The evidence can also be related to the issue of economic resources and welfare state policy implications in the OECD economies, and these countries are not significantly affected by uncertainty shocks related to pandemics.

Furthermore, the control variables' significant effects on the fertility rates. For instance, the lagged per capita GDP is positively associated with the fertility rate. The related coefficients are statistically significant at the 5% level at least. The lagged life expectancy decreases the fertility rate, and their coefficients are also statistically significant at the 1% level. The lagged human capital is positively related to the fertility rate. The coefficients are statistically significant at the 1% level for all countries and non-OECD economies; however, the coefficients are statistically insignificant in OECD economies. This evidence suggests that the effects of pandemics shocks on fertility are essential even though various controls are included.

Our findings are based on the coefficients of the level of WPUI, and the lagged WPUI shows no significant effect on fertility. This evidence related to the issue that the impact of the uncertainty shock on fertility behavior is mainly valid in the short run, as previous papers [e.g., (24, 66)] suggested.

### Change of pandemics-related uncertainty shocks

Table 3 reports the findings of the fixed-effects estimations for Model (2), which focus on the difference between the fertility rate and the first difference in the WPUI.

**Table 3:** Fixed-effects estimations for Model II (1996–2019).

**Regressors**	**All countries**	**All countries**	**Non-OECD**	**Non-OECD**	**OECD**	**OECD**
Log per capita GDP_t−1_	0.023*** (0.007)	0.023*** (0.007)	0.021*** (0.007)	0.022*** (0.007)	0.054*** (0.017)	0.053*** (0.020)
Life expectancy_t−1_	−0.003*** (0.001)	−0.003*** (0.001)	−0.003*** (0.001)	−0.003*** (0.001)	−0.008*** (0.002)	−0.009*** (0.003)
Index of human capital _t−1_	0.091*** (0.017)	0.081*** (0.017)	0.104*** (0.020)	0.094*** (0.019)	0.054 (0.038)	0.052 (0.041)
ΔWPUI_t_	−0.285*** (0.076)	–	−0.243*** (0.069)	–	−0.820*** (0.198)	–
ΔWPUI_t−1_	–	−0.137 (0.096)	–	−0.127 (0.077)	–	−0.254 (0.395)
Intercept	−0.204*** (0.064)	−0.180*** (0.062)	−0.216*** (0.065)	−0.196*** (0.063)	−0.043 (0.155)	0.027 (0.168)
Observation	2,722	2,646	2,024	1,932	748	714
Countries	126	126	92	92	34	34
*R*^2^ (Within)	0.109	0.092	0.157	0.138	0.031	0.028

The dependent variable is ΔFertility Rate_t_. The standard errors are in ( ).

^***^p < 0.01.

Again, Columns 1 and 2 provide the findings for all (126) countries from 1996 to 2019. The first column reports the results of the current WPUI, and the second column provides the findings of the lagged WPUI. In addition, the results of 92 non-OECD economies are reported in Columns 3 and 4. The results of 34 OECD economies are compared in Columns 5 and 6.

In all countries, the coefficient of the current WPUI is −0.285, and it is significant at the 1% level. Similarly, the coefficient of the current WPUI is −0.243 for the non-OECD economies, and it is significant at the 1% level. Also, the current WPUI is −0.82 for the OECD economies and is statistically significant at the 1% level. In addition, the coefficients of the lagged WPU are negative, but they are statistically insignificant. These findings show that pandemics-related uncertainty has an adverse and temporary effect on the fertility rate in all countries.

On the other hand, the significant effects of the controls on the fertility rates are observed. Specifically, the lagged per capita GDP is positively related to the fertility rate. Their coefficients are statistically significant at the 1% level. The lagged life expectancy reduces the fertility rate, and the related coefficients are also statistically significant at the 1% level. The lagged human capital increases the fertility rate. The corresponding coefficients are statistically significant at the 1% level for all countries and the non-OECD economies; however, the coefficients are statistically insignificant in the OECD economies. These results show that pandemics hurt fertility, mostly in poor or developing economies. These findings indicate that the effects of pandemics shocks on fertility are crucial when various controls are included. According to most papers in the empirical literature, the negative impact of COVID-19 is mainly valid in developed economies. However, according to Aassve et al. (66), the COVID pandemic also increased economic losses and uncertainty in the Low- and Middle-income economies. This negative impact occurs in transition economies and urban areas of developing economies and causes the decline of the population size in developing economies.

## Robustness checks

### Persistent fertility rates and level of pandemics-related uncertainty shocks

Table 4 provides the results of the fixed-effects estimations for Model (3), which focus on the fertility rate and the level of the WPUI.

**Table 4:** Persistent fertility rates: fixed-effects estimations for Model I (1996–2019).

**Regressors**	**All countries**	**All countries**	**Non-OECD**	**Non-OECD**	**OECD**	**OECD**
Fertility rate_t−1_	0.966*** (0.005)	0.966*** (0.005)	0.970*** (0.005)	0.970*** (0.005)	0.936*** (0.014)	0.936*** (0.014)
Log per capita GDP_t−1_	0.021*** (0.008)	0.021*** (0.008)	0.019*** (0.007)	0.019*** (0.007)	0.064*** (0.018)	0.064*** (0.018)
Life expectancy_t−1_	−0.005*** (0.001)	−0.005*** (0.001)	−0.005*** (0.001)	−0.005*** (0.001)	−0.007** (0.003)	−0.007** (0.003)
Index of human capital_t−1_	0.080*** (0.017)	0.080*** (0.017)	0.088*** (0.019)	0.088*** (0.019)	0.016 (0.044)	0.015 (0.044)
WPUI_t_	−0.356*** (0.133)	–	−0.316*** (0.139)	–	−0.787 (0.550)	–
WPUI_t−1_	–	−0.092 (0.137)	–	−0.064 (0.136)	–	−0.082 (0.126)
Intercept	0.083 (0.074)	0.082 (0.074)	0.065 (0.085)	0.064 (0.085)	−0.049 (0.157)	−0.053 (0.157)
Observation	2,722	2,722	2,024	2,024	748	748
Countries	126	126	92	92	34	34
*R*^2^ (Within)	0.988	0.988	0.991	0.991	0.896	0.896

The dependent variable is Fertility Rate_t_. The standard errors are in ( ).

^***^p < 0.01.

^**^p < 0.05.

Again, Columns 1 and 2 report the results for 126 countries from 1996 to 2019. The first column provides the findings of the current WPUI. The second column reports the results of the lagged WPUI. In addition, the results of 92 non-OECD economies are provided in Columns 3 and 4. In comparison, the findings of 34 OECD economies are reported in Columns 5 and 6.

In all countries, the coefficient of the current WPUI is −0.356, and it is significant at the 1% level. Similarly, the coefficient of the current WPUI is −0.316 for the non-OECD economies, and it is significant at the 1% level. However, the current WPUI is −0.787 for the OECD economies, which is statistically insignificant. Moreover, the coefficients of the lagged WPU are positive, but they are statistically insignificant. These results indicate that the pandemics-related uncertainty has an adverse and temporary effect on fertility behavior in developing economies.

It is also noteworthy that the controls statistically affect the fertility rate. Specifically, the lagged fertility rate is statistically significant at 1%, indicating a substantial persistency in fertility decisions. The lagged per capita GDP is positively related to the fertility rate. The related coefficients are statistically significant at the 1% level. The lagged life expectancy decreases the fertility rate, and the associated coefficients are also statistically significant at the 1% level. The lagged human capital spurs the fertility rate. The related coefficients are statistically significant at the 1% level for all countries and the non-OECD economies; however, the coefficients are statistically insignificant in the OECD economies. These results show that the effects of pandemics shocks on fertility are essential when various controls are included.

### Persistent fertility rates and change of pandemics-related uncertainty shocks

Table 5 reports the findings of the fixed-effects estimations for Model (4), which focus on the level of the fertility rate and the difference in the WPUI.

**Table 5:** Persistent fertility rates: fixed-effects estimations for Model II (1996–2019).

**Regressors**	**All countries**	**All countries**	**Non-OECD**	**Non-OECD**	**OECD**	**OECD**
Fertility rate_t−1_	0.966*** (0.005)	0.964*** (0.005)	0.970*** (0.005)	0.968*** (0.005)	0.936*** (0.015)	0.936*** (0.015)
Log per capita GDP_t−1_	0.021*** (0.008)	0.022*** (0.007)	0.019** (0.008)	0.020** (0.007)	0.064*** (0.018)	0.061*** (0.019)
Life expectancy_t−1_	−0.005*** (0.001)	−0.006*** (0.001)	−0.005*** (0.001)	−0.005*** (0.001)	−0.007*** (0.002)	−0.007*** (0.002)
Index of human capital_t−1_	0.080*** (0.017)	0.071*** (0.016)	0.088*** (0.019)	0.079*** (0.018)	0.016 (0.044)	0.015 (0.048)
ΔWPUI_t_	−0.260*** (0.072)	–	−0.223*** (0.065)	–	−0.765*** (0.193)	–
ΔWPUI_t−1_	–	−0.126 (0.094)	–	−0.117 (0.074)	–	−0.248 (0.407)
Intercept	0.082 (0.074)	0.120* (0.072)	0.064 (0.085)	0.100 (0.082)	−0.052 (0.156)	0.008 (0.163)
Observation	2,722	2,646	2,024	1,932	748	748
Countries	126	126	92	92	34	34
*R*^2^ (Within)	0.988	0.987	0.991	0.991	0.896	0.888

The dependent variable is Fertility Rate_t_. The standard errors are in ( ).

^***^p < 0.01.

^**^p < 0.05.

^*^p < 0.10.

Again, Columns 1 and 2 provide the findings for 126 countries from 1996 to 2019. The first column provides the results of the current WPUI. The second column reports the findings of the lagged WPUI. In addition, the findings of 92 non-OECD economies are reported in Columns 3 and 4. The results of 34 OECD economies are compared in Columns 5 and 6.

In all countries, the coefficient of the current WPUI is −0.26. The current WPUI is −0.223 for non-OECD and −0.765 for OECD economies. All of these coefficients are statistically significant at the 1% level. Furthermore, the coefficients of the lagged WPU are positive, but they are statistically insignificant. These findings show that the pandemics-related uncertainty has an adverse and temporary effect on fertility decisions in all countries.

It is also noteworthy to note that the significant effects of the controls on the fertility rates are found. For instance, the lagged fertility rate is statistically significant at 1%, meaning a considerable persistency in fertility behavior. The lagged per capita GDP is positively related to the fertility rate. The related coefficients are statistically significant at the 5% level at least. The lagged life expectancy reduces the fertility rate, and the associated coefficients are also statistically significant at the 1% level. The lagged human capital increases the fertility rate. The related coefficients are statistically significant at the 1% level for all countries and the non-OECD economies; however, the coefficients are statistically insignificant in the OECD economies. These findings again indicate that the effects of pandemics shocks on fertility are essential even though various controls are included.

In short, various empirical model estimations indicate that pandemics-related uncertainty decreases the fertility rate, and the effect is temporary. Per capita GDP and human capital increase fertility, but life expectancy is negatively associated with fertility. These results are statistically significant for developing economies in all models.

The results in Tables 3, 5 indicate that the effect size of difference of WPUI among OECD is greater than that among non-OECD countries. This evidence is in line with the findings of most papers in the empirical literature, i.e., the negative impact of COVID-19 is mainly valid in developed countries. This evidence is primarily related to the issue of demographic transition and age distribution (population aging) in different countries. In developed countries, where the negative impact of COVID-19 is higher, the demographic transition is slow, and population aging is a more severe problem than the developing countries.

## Conclusion and recommendations

### Conclusion

This paper examines the effects of pandemics-related uncertainty on fertility behavior in the panel dataset of 126 countries from 1996 to 2019. For this purpose, we use the WPUI introduced by Ahir et al. (23) to capture the pandemics-related uncertainty. The WPUI measure models pandemics' uncertainty, which is exogenous to the fertility behavior. The novel empirical evidence is that the WPUI reduces the fertility rate. The empirical results show that per capita income and human capital promote the fertility rate, but a higher life expectancy decreases fertility. These results are robust to estimating different models.

### Limitations and recommendations

Our evidence from the WPUI may also explain why the rise in uncertainty during COVID-19 resulted in fertility decline. It suggests that fertility is negatively associated with business cycles due to increased uncertainty. However, our results are limited to the panel data across various countries. Note that the sample is the main limitation of our findings since most WPUI values are zero in our case. The COVID-19 pandemic can change the dynamics of uncertainty shocks on fertility at this stage. For instance, Wilde et al. (24) use Google Trend searches data to show that the COVID-19 pandemic reduces fertility due to the rising unemployment (a measure of uncertainty shock). Therefore, future papers can use different indicators of uncertainty shocks to explain the difference in the fertility rates across countries during the COVID-19 pandemic era.

It is also essential to indicate that our findings are valid, with a limited number of control variables included. Therefore, future papers include more control variables in the model estimations. Finally, future articles can use individual microdata to analyse pandemics-related uncertainty shocks' effects on the fertility rate.

## Data availability statement

Publicly available datasets were analyzed in this study. This data can be found here: https://databank.worldbank.org/source/world-development-indicators; https://www.rug.nl/ggdc/productivity/pwt/?lang=en; https://worlduncertaintyindex.com/data/.

## Author contributions

YW: writing introduction and literature review. GG: collection of the data and empirical estimations. CL: writing the results and conclusion and proofreading. All authors contributed to the article and approved the submitted version.

## Conflict of interest

The authors declare that the research was conducted in the absence of any commercial or financial relationships that could be construed as a potential conflict of interest.

## Publisher's note

All claims expressed in this article are solely those of the authors and do not necessarily represent those of their affiliated organizations, or those of the publisher, the editors and the reviewers. Any product that may be evaluated in this article, or claim that may be made by its manufacturer, is not guaranteed or endorsed by the publisher.

## Figures and Tables

**Table A1:** 126 Economies in the dataset.

Albania, Algeria, Angola, Argentina, Armenia, Australia, Austria, Bangladesh, Belgium, Benin, Bolivia, Botswana, Brazil, Bulgaria, Burkina Faso, Burundi, Cambodia, Cameroon, Canada, Central African Republic, Chile, China, Colombia, Congo DR, Congo Republic, Costa Rica, Côte d'Ivoire, Croatia, Czech Republic, Denmark, Dominican Republic, Ecuador, Egypt, El Salvador, Ethiopia, Finland, France, Gabon, the Gambia, Germany, Ghana, Greece, Guatemala, Haiti, Honduras, Hong Kong SAR (China), Hungary, India, Indonesia, Iran, Iraq, Ireland, Israel, Italy, Jamaica, Japan, Jordan, Kazakhstan, Kenya, Korea Republic, Kuwait, Kyrgyz Republic, Laos, Latvia, Lesotho, Liberia, Lithuania, Madagascar, Malawi, Malaysia, Mali, Mauritania, Mexico, Moldova, Mongolia, Morocco, Mozambique, Myanmar, Namibia, Nepal, the Netherlands, New Zealand, Nicaragua, Niger, Nigeria, Norway, Pakistan, Panama, Paraguay, Peru, the Philippines, Poland, Portugal, Qatar, Romania, Russia, Rwanda, Saudi Arabia, Senegal, Sierra Leone, Singapore, Slovak Republic, Slovenia, South Africa, Spain, Sri Lanka, Sudan, Sweden, Switzerland, Tajikistan, Tanzania, Thailand, Togo, Tunisia, Turkey, Uganda, Ukraine, the United Arab Emirates, the United Kingdom, the United States, Uruguay, Venezuela, Vietnam, Yemen, Zambia, and Zimbabwe.
